# Effect of a 20-week physical activity intervention on selective attention and academic performance in children living in disadvantaged neighborhoods: A cluster randomized control trial

**DOI:** 10.1371/journal.pone.0206908

**Published:** 2018-11-08

**Authors:** Stefanie Gall, Larissa Adams, Nandi Joubert, Sebastian Ludyga, Ivan Müller, Siphesihle Nqweniso, Uwe Pühse, Rosa du Randt, Harald Seelig, Danielle Smith, Peter Steinmann, Jürg Utzinger, Cheryl Walter, Markus Gerber

**Affiliations:** 1 Department of Sport, Exercise and Health, University of Basel, Basel, Switzerland; 2 Department of Human Movement Science, Nelson Mandela University, Port Elizabeth, South Africa; 3 University of Basel, Basel, Switzerland; 4 Swiss Tropical and Public Health Institute, Basel, Switzerland; TNO, NETHERLANDS

## Abstract

**Objectives:**

To evaluate the effect of a 20-week school-based physical activity intervention program on academic performance and selective attention among disadvantaged South African primary school children.

**Design:**

Cluster randomized control trial.

**Methods:**

The study cohort included 663 children from eight primary schools, aged 8–13 years. Data assessment took place between February 2015 and May 2016 following the implementation of a 20-week school-based physical activity program. The d2 test was employed to assess selective attention, while the averaged end-of-year school results (math, life skills, home language, and additional language) were used as an indicator of academic performance. Physical fitness was assessed using the 20-m shuttle run test (VO_2_ max) and grip strength tests. We controlled for cluster effects, baseline scores in selective attention or academic performance, and potential confounders, such as children’s age, gender, socioeconomic status, self-reported physical activity (as determined by a pre-tested questionnaire), body mass index, hemoglobin (as a proxy for anemia, as measured by blood sampling), and soil-transmitted helminth infections (as assessed by the Kato-Katz technique).

**Results:**

Our multivariate analysis suggested that the physical activity intervention had a positive effect on academic performance (p = 0.032), while no effect was found on selective attention (concentration performance; p = 0.469; error percentage; p = 0.237). After controlling for potential confounders, the physical activity condition contributed to the maintenance of academic performance, whereas a decrease was observed in learners in the control condition. Furthermore, physically active and fit children tend to have better concentration performance (CP) than their less fit peers (self-reported activity; p<0.016, grip strength; p<0.009, VO_2_ max p>0.021).

**Conclusion:**

A 20-week physical activity intervention contributes to the maintenance of academic performance among socioeconomically deprived school children in South Africa. School administrators should ensure that their school staff implements physical activity lessons, which are a compulsory component of the school by the curriculum.

## Introduction

Physical activity is widely accepted to be an important feature in the promotion of health and well-being [1]. In view of the mounting evidence of health-related benefits of regular physical activity among children [2–4], concerns have been raised about decreasing physical activity and fitness levels in children and adolescents [5, 6]. New research reveals that regular physical activity not only contributes to improved physical health [7], but also has a beneficial effect on children’s cognitive functioning, such as executive function [8], attention [9], and academic performance [10], all of which are important conditions for gains in academic performance. Yet, physical education has been neglected in many low- and middle-income countries (LMICs), while more time is being allocated to academic subjects [11]. In South Africa, some teachers and parents believe that participation in physical activity might interfere with learners’ academic success [12]. Educators often teach several subjects and do not feel confident enough to systematically instruct in sports and exercise as part of a school program. Indeed, they do not feel able to offer attractive and didactically well-conducted physical education classes. Furthermore, some of them lack the motivation to be physically active. Hence, physical education is not being taught in most public schools. As a result, a considerable number of children do not engage in the recommended daily 60 min of moderate-to-vigorous intensity physical activity [13]. According to Walter [14], this might be one of the key contributors to the increasing level of physical inactivity among South African school children.

While low physical activity levels constitute an important issue from a public health perspective [15], children growing up and living in low socioeconomic environments are faced with a multitude of challenges that may jeopardize their health and well-being [16]. These challenges include insufficient hygiene, lack of clean water, and inadequate sanitation, factors that all favor helminth and intestinal protozoa infection [17]. This, in turn, can cause abdominal pain, diarrhea, growth retardation, anemia, cognitive impairment, poor academic performance and reduced physical fitness [18, 19]. Of note, the effects of mass deworming on children’s cognition and school performance remain ambiguous [20]. Another potentially harmful effect of living in deprived environments is the commonly limited access to health care in the absence of universal health coverage. Consequently, increased risk of illness causes more frequent school absenteeism, and hence, reduced academic exposure [21]. Poor nutrition and repeated infection causes stunting, which in turn has been found to be associated with motor development problems and poor cognitive development resulting in low intelligence quotient [22]. Taken together, these factors can obstruct children’s ability to process information, concentrate, and focus on academic work [21].

While researchers have pointed toward the potential of physical activity and physical education to increase cognitive performance and academic performance [23–25], there is a paucity of studies on the effects of school-based physical activity intervention programs on children’s schooling outcomes in disadvantaged areas from LMICs. To fill this gap, the aim of the present study was to examine the impact of a 20-week school-based physical activity intervention on selective attention and academic performance on children attending schools in disadvantaged areas in Port Elizabeth, South Africa.

## Materials and methods

### Ethics statement

The study was approved by the ethics committee of Northwestern and Central Switzerland (EKNZ; reference no. 2014–179, approval date: 17 June 2014), the NMU Ethics Committee (study number H14-HEA-HMS002, approval date: 4 July 2014), the Eastern Cape Department of Health (approval date: 7 November 2014), and the Eastern Cape Department of Education (approval date: 3 August 2014). The study is registered at ISRCTN registry under controlled-trials.com (unique identifier: ISRCTN68411960, registration date: 1 October 2014). Written informed consent was obtained from the parents/legal guardians of children, while children assented orally. Details regarding the information provided to eligible participants and their parents/legal guardians, as well as the inclusion and exclusion criteria can be found in the study protocol [26]. All procedures were in line with ethics principles described in the 1964 Declaration and its later amendments. Participation was voluntary, and children could withdraw at any time without further obligation.

### Study area, school selection, and randomization

The DASH (Disease, Activity, and Schoolchildren’s Health) study was carried out in quintile three schools, located in disadvantaged communities of Port Elizabeth, South Africa. All quintile three primary schools in the Port Elizabeth district (n = 103) were invited to participate in the study. South African public schools are classified into five groups, with quintile five standing for the least poor and quintile one standing for the poorest. The quintiles are determined through the national poverty table, prepared by the Treasury. Areas are being ranked on the basis of income levels, dependency ratios, and literacy rates in the area. The quintile ranking of a school determines the no-fee status of the school and also the amount of money that a school receives, with the poorest schools receiving the greatest per-learner allocation [27].

From the 103 quintile three schools, 25 schools expressed an interest in the form of a written response. Those 25 schools were invited to an information sharing meeting, and 15 schools were represented, whereas 10 schools declined to attend the meeting. The final eight schools were selected based on (i) large grade four classes (n >100); (ii) geographical location; (iii) representation of the various target communities (black and colored inhabitants); and (iv) commitment to support the project activities. The seven schools that were excluded had all numbers <100 in grade four. Due to financial constraints, logistics, and limited manpower, we were able to implement the physical activity intervention program only in three schools. From the final eight schools, the DASH core team therefore randomly selected three intervention and five control schools, on the basis of a computer-generated random number list. A computer generated random number list was also used to allocate the intervention and control schools to one of five intervention/control conditions (see Table 1).

**Table 1:** Intervention measures at the eight primary schools in Port Elizabeth, South Africa carried out between February 2015 and May 2016.

Intervention	IG	CG
Physical activity	School 1	
Physical activity + health education	School 2	
Physical activity + health education + nutrition	School 3	
[No physical activity] + health education + nutrition		School 4
[No physical activity]		Schools 5, 6, 7, and 8

IG, intervention group; CG, control group.

### Participants

**Fig 1:**
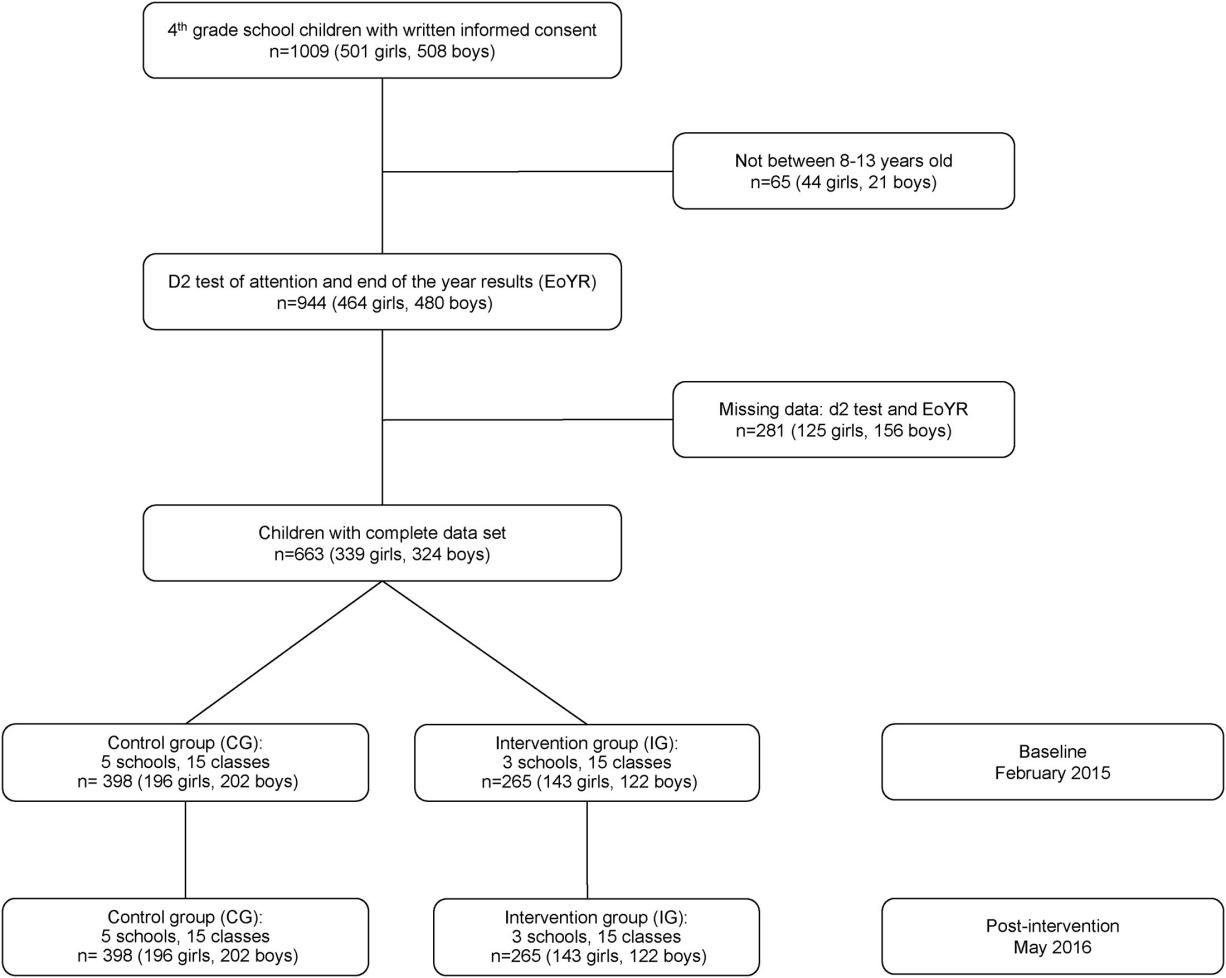
Participant flow chart. Notes: EoYR: end of the year results, average of the four subjects home language, additional language, mathematics, and life skills.

As shown in Fig 1, 1009 grade four primary school children were assessed at baseline in February 2015. Data sets with complete records in the main outcome variables concentration performance (CP), error percentage (E%), and end of the year results (EoYR) were available for 663 learners at post-intervention in May 2016, after including only children aged 8–13 years. The mid-follow-up is not included in the present paper.

### Procedures

**Fig 2:**
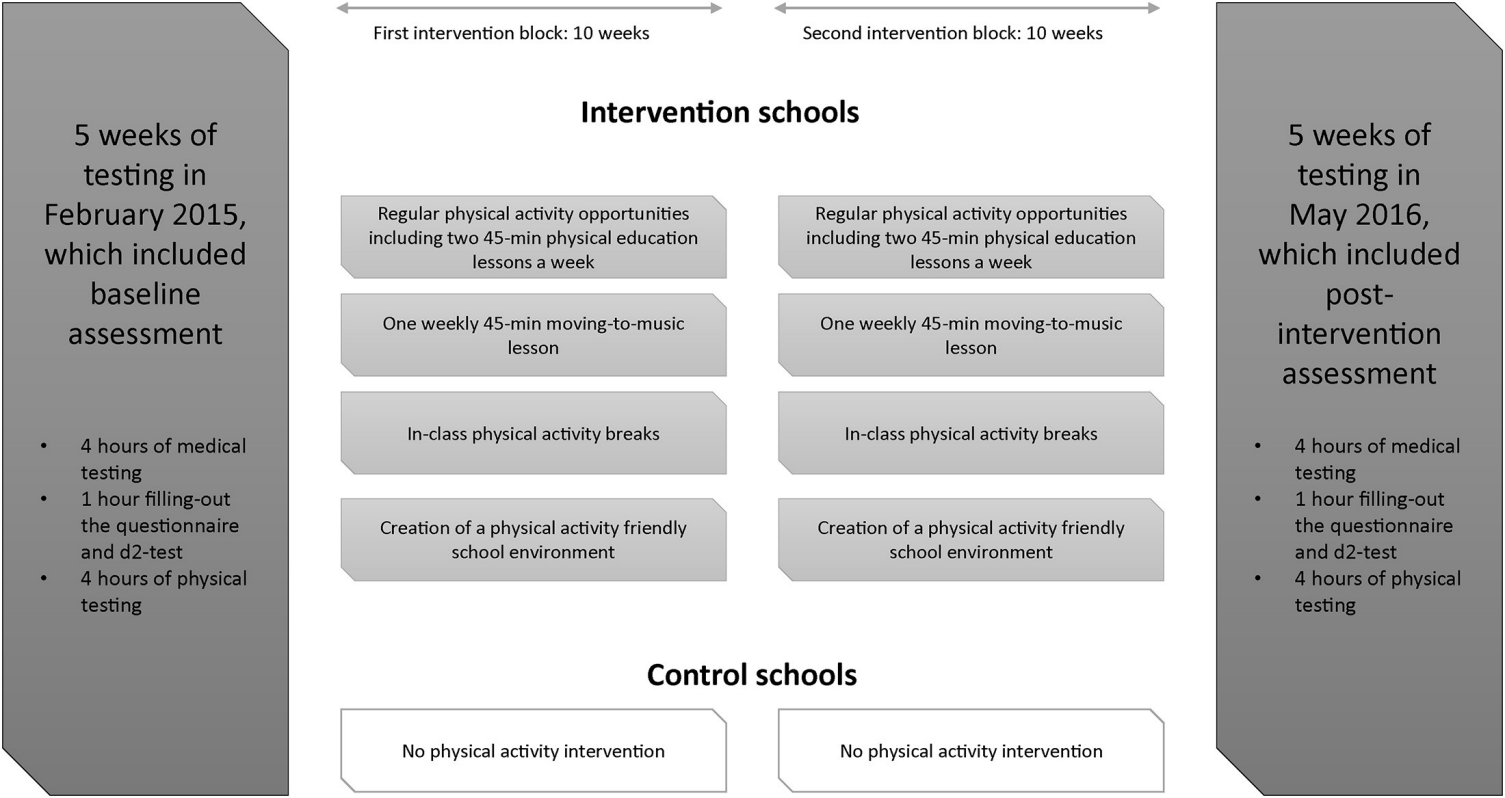
Overview of study design and the 20-week physical activity intervention, Port Elizabeth, South Africa carried out between February 2015 and May 2016.

An overview of the study design is provided in Fig 2. Recruitment started in September 2014, and the practical intervention study took place between February 2015 and May 2016. The study length was restricted to 15 months due to financial reasons.

At the beginning of the study, project information sessions with principals, teachers, parents, and school governing bodies were held. Workshops with life orientation teachers and class teachers were organized to discuss the materials and information provided during the intervention.

### Multi-dimensional physical activity intervention

The multi-dimensional physical activity intervention started after the baseline assessment and was carried out during official school lessons. The lessons were taught in collaboration with the life orientation teachers, and moving-to-music lessons were led by students from the Nelson Mandela University (NMU). The control group followed their normal schedule, with physical education following the routine curriculum. The physical activity intervention module included four parts: (i) regular physical activity classes including two physical education lessons per week; (ii) one weekly moving-to-music class; (iii) regular in-class activity breaks incorporated into the main school curriculum; and (iv) school infrastructure adaptation to create a low cost “physical activity friendly” environment (Fig 2). The physical activity intervention was implemented at three schools, whereas five schools did not receive the physical activity intervention. Furthermore, in two schools, a nutrition intervention consisting of classroom-based lessons to help increase the awareness of the importance of healthy nutrition were held. At three schools, health education lessons were held to increase children’s awareness for intestinal parasite infections (Table 1).

### Selective attention

Main outcome variables were selective attention and academic performance. Children’s selective attention capacity was measured with the d2 test, developed by Brickenkamp et al. [28]. The d2 test determines the capacity to pay attention to one stimulus/fact, while suppressing distractor letters. The d2 attention test is a paper and pencil letter-cancellation test and was performed in groups of 20–25 children, during the first school lesson of the day in a quiet room. Detailed test procedures can be found in the test manual [28] and in a previous publication on selective attention [29]. Selective attention capacity was operationalized via two indicators: (i) E% and (ii) CP. E% is calculated as percentage of errors relative to the total number of items processed and reflects a measure of accuracy (precision and thoroughness). CP represents a measure of overall concentration ability and performances and is calculated by subtracting the number of incorrectly marked characters from the number of correctly marked characters. Both E% and CP were chosen as outcome measures because they are tamper-resistant.

### Academic performance

Children’s academic performance was operationalized using routine EoYR. Note that EoYR are based on the average from four school subjects: (i) home language (Xhosa or Afrikaans); (ii) first additional language (English); (iii) mathematics; and (iv) life skills. Learners’ achievement in each subject is graded on a 7-point scale ranging from 1 (0–29%, “not achieved”) to 7 (80–100%; “outstanding achievement”), with a rating of 4 (50–59%) reflecting “adequate achievement”. In order to control for potential confounders, a range of additional variables was assessed and considered in the present article.

### Physical fitness tests

To control for children’s cardiorespiratory fitness, the 20-m shuttle run test was applied using the test protocol by Léger et al. [30]. The fully completed laps were noted when a child was unable to follow the required pace in two consecutive intervals. The level at which the child failed to reach the cut-off line during the 20-m shuttle run test was used to calculate an estimate of maximal oxygen uptake (VO_2_ max), which was then adjusted for age and gender [30].

Upper body strength was determined by the grip strength test [31]. The Saehan hydraulic hand dynamometer (MSD Europe BVBA; Tisselt, Belgium) was used for this purpose. The trials were recorded to the nearest 1 kg and averaged. The detailed test description of the 20-m shuttle run and the grip strength test can be found in the study protocol [26].

### Self-reported physical activity

To consider children’s habitual physical activity levels, children were asked to self-report their physical activity levels using a single-item question from the Health-Behavior of School-Aged Children (HBSC) study [32]: “Over the past 7 days (1 week), on how many days were you physically active for a total of at least 60 min (1 hour) a day?” The answering options ranged from zero days to seven days. This question is based on the recommendation for physical activity among young people stating that children and youth aged 6–17 years should perform at least 60 min of moderate-to-vigorous intensity physical activity per day [33].

### Anthropometric indicators

To control for anthropometric factors, children’s height and weight were assessed. Body weight was measured once to the nearest 0.1 kg (using a Micro T7E electronic platform scale, Optima Electronics; George, South Africa). The body height of each child was assessed to the nearest 0.1 cm with a Seca Stadiometer (Surgical SA; Johannesburg, South Africa). Body weight and height values were used to calculate body mass index (BMI), defined as weight (in kg)/height^2^ (in m^2^) and to reflect BMI-for-age (BAZ) and height-for-age (HAZ), respectively, both stratified by gender. The BAZ and HAZ were calculated using the World Health Organization (WHO) growth reference [34]. The gender-adjusted HAZ scores were used as an indicator for stunting [35].

### Hemoglobin

Hemoglobin (Hb) concentration was measured once to the nearest 0.1 g/l with the Hemocue HB 301 System (HemoCue AB; Ängelholm, Sweden) as a proxy for anemia. After swabbing the child’s fingertip with alcohol, a field worker pricked the fingertip with a safety lancet and squeezed gently to obtain blood. The first drop was wiped away with the alcohol swab and the second drop was collected for testing with a micro cuvette.

### Soil-transmitted helminth infections

To account for the potential influence of soil-transmitted helminths on selective attention and academic performance, stool samples were collected and analyzed. In brief, school children were instructed and informed in the course of a morning during a school day to return the distributed stool containers with a small portion of their own morning stool the following day. The diagnostic work-up of stool samples was done on the day of collection. From each stool sample duplicate 41.7 mg Kato-Katz thick smears were prepared [36]. Experienced laboratory technicians independently read the slides under a microscope, counted the number of helminth eggs, and recorded them for each species separately. To obtain a proxy for infection intensity, the soil-transmitted helminth egg counts were multiplied by a factor of 24 to express the number of eggs per gram (EPG) of stool [37].

### Socioeconomic status

Children filled out a questionnaire pertaining to household-level living standards, such as infrastructure and housing characteristics (e.g., wall and roof type, number of bedrooms) and the ownership of three durable assets (e.g., refrigerator) to determine their socioeconomic status (SES) with nine questions. The binary items (1 = high quality, available; 0 = poor quality, not available) were summarized to build an overall index, with lower scores reflecting lower SES. Similar measures have been validated and established in previous research [38].

### Test sequence

Children were assessed class wise and the test procedure lasted for two full school days at each of the eight schools (baseline and post-intervention). In a first step, children completed the medical testing including the anthropometric indicators, followed by the grip strength test. On the second day, children completed the d2 test of attention and filled in a questionnaire for determining their SES. Then, children’s cardiorespiratory fitness was assessed with the 20-m shuttle run test (Fig 2).

## Statistical analysis

Data were double-entered and validated using EpiData version 3.1 (EpiData Association; Odense, Denmark), and merged into a single data file. Statistical analyses were performed with STATA version 13.0 (STATA; College Station, United States of America) and with SPSS version 23 (IBM Corporation; Armonk, United States of America) for Windows.

Sample size calculation is described in the study protocol [26]. In brief, sample size was based on the prevalence of soil-transmitted helminth infections at baseline, taking into account clustering within schools and classes as well as loss to follow-up.

Descriptive statistics are provided as frequencies (n) and percentages (%) for categorical variables, and means (M) and 95% confidence interval (CI) for metric variables, separately for the total sample, and learners of the intervention group (IG) and control group (CG).

To determine whether a 20-week school-based physical activity intervention had an effect on selective attention (E% and CP) and academic performance (EoYR), three separate mixed linear regression models were employed with random intercepts for school classes, in order to adjust for cluster effects. This was facilitated using the *multilevel mixed effects linear regression* procedure (covariance structure = independent) in STATA version 13.0 (STATA; College Station, United States of America). In all three regression analyses, indicators of selective attention and academic performance at post-intervention were used as outcome/dependent variables. Before testing the effect of the intervention condition (IG *versus* CG), all regression analyses were controlled for baseline levels of selective attention and academic performance and potential confounders (gender, stunting, anemia, intestinal protozoa and soil-transmitted helminth infections, age, BMI, SES, self-reported physical activity, grip strength, and VO_2_ max).

To interpret the findings, the unstandardized Beta coefficients and the 95% confidence intervals (CIs) are displayed. Statistical significance level was set at p<0.05 across all analyses. Some missing data occurred in the covariates in less than 5% of all possible data entries. Detailed missing data inspection revealed no systematic missing data pattern or significant correlations. Hence, it was decided not to impute missing data and the mixed linear regression models were based on data of children with complete data records.

## Results

Table 2 summarizes the descriptive statistics of selective attention and academic performance (at baseline and post-intervention) and of the potential confounders (at baseline), separately for the total sample, IG, and CG. A first inspection revealed that the descriptive measures indicate few noticeable differences between the IC and CG at baseline. The groups seemed to differ in levels of self-perceived physical activity (IG: M = 4.03; 95% CI: 3.73 to 4.33] *versus* CG: M = 2.97; 95% CI: 2.74 to 3.21), concentration (CP) (IG: M = 51.33; 95% CI: 47.68 to 54.98 *versus* CG: M = 58.34; 95% CI: 55.74 to 60.94) and academic performance (EoYR) (IG: M = 3.90; 95% CI: 3.75 to 4.06 *versus* CG: M = 4.93; 95% CI: 4.80 to 5.06).

**Table 2:** Descriptive statistics of all variables at baseline in February 2015 and outcome/dependent variables also at post-intervention in May 2016, Port Elizabeth, South Africa.

	**Baseline**		
	**Total**	**CG**	**IG**^a^
**Potential confounder**	n (%)	n (%)	n (%)
Girls	339 (51)	196 (49)	143 (54)
Stunted^b^	57 (9)	26 (7)	31 (12)
Anemic^c^	106 (17)	65 (17)	41 (16)
Infected with Intestinal protozoa^d^	84 (14)	41 (11)	43 (18)
Infected with soil-transmitted helminths^e^	172 (28)	82 (22)	90 (38)
**Potential confounder**	M (95% CI)	M (95% CI)	M (95% CI)
Age in years	9.24 (9.19 to 9.30) n = 663	9.22 (9.14 to 9.29) n = 398	9.28 (9.20 to 9.36) n = 265
Height in cm	132.37 (131.85 to 132.91) n = 660	132.72 (132.06 to 133.38) n = 398	131.85 (130.96 to 132.74) n = 262
Weight in kg	30.21 (29.61 to 30.81) n = 660	30.34 (29.57 to 31.10) n = 398	30.01 (29.04 to 30.99) n = 262
BMI in kg m^-2^	17.06 (16.82 to 17.31) n = 660	17.06 (16.75 to 17.38) n = 398	17.07 (16.69 to 17.45) n = 262
SES^f^	7.55 (7.40 to 7.71) n = 660	7.53 (7.33 to 7.74) n = 397	7.58 (7.34 to 7.82) n = 263
Score of self-perceived physical activity^g^	3.41 (3.22 to 3.59) n = 658	2.98 (2.75 to 3.21) n = 393	4.03 (3.73 to 4.33) n = 265
Grip strength in kg	11.81 (11.56 to 12.05) n = 618	11.87 (11.55 to 12.20) n = 371	11.70 (11.33 to 12.07) n = 247
VO_2_ max in ml kg^-1^ min^-1^	49.19 (48.85 to 49.52) n = 597	49.52 (49.09 to 49.95) n = 356	48.69 (48.17 to 49.21) n = 241
		**Baseline**	
	Total (N = 663)	CG (n = 398)	IG^a^ (n = 265)
**Selective attention and academic performance**	M (95% CI)	M (95% CI)	M (95% CI)
CP in points^h^	55.54 (53.39 to 57.68)	58.34 (55.74 to 60.94)	51.33 (47.68 to 54.98)
E%^i^	15.74 (14.82 to 16.66)	14.74 (13.62 to 15.87)	17.24 (15.68 to 18.81)
EoYR^j^	4.52 (4.42 to 4.63)	4.93 (4.80 to 5.06)	3.90 (3.75 to 4.06)
		**Post-intervention**	
	Total (N = 663)	CG (n = 398)	IG (n = 265)
**Selective attention and academic performance**	M (95% CI)	M (95% CI)	M (95% CI)
CP in points^h^	98.03 (95.30 to 100.76)	100.27 (96.85 to 103.70)	94.66 (90.18 to 99.15)
E%^i^	8.08 (7.31 to 8.84)	7.29 (6.36 to 8.22)	9.26 (7.95 to 10.57)
EoYR^j^	4.10 (4.02 to 4.18)	4.26 (4.16 to 4.36)	3.87 (3.75 to 4.00)

^a^ School children from the intervention group took part in a 20-week physical activity intervention program, as described in Fig 2

^b^ Stunting is defined as HAZ score ≤-2, (1 = stunted, 0 = normal)

^c^ Anemic is defined as hemoglobin concentration in blood ≤114 g/l, (1 = anemic, 0 = normal)

^d^ Infected with one or two intestinal parasite species (*Cryptosporidium* spp. and/or *Giardia intestinalis*) (1 = infected, 0 = not infected)

^e^ Infected with one or two soil-transmitted helminth species (*Ascaris lumbricoides* and/or *Trichuris trichiura*) (1 = infected, 0 = not infected)

^f^ Socioeconomic status (SES) measured by ownership and housing related questions on a scale from 0–9 points (0 = lowest score, 9 = highest score)

^g^ Score of self-reported physical activity for the past 7 days on a scale from 0–7 (0 = lowest score, 7 = highest score)

^h^ Concentration performance: total number of correctly cancelled minus total number incorrectly cancelled characters (d2 test of attention)

^i^ Percentage of errors: total number of errors divided by the total number of characters processed (d2 test of attention)

^j^ Average of the four subjects home language, additional language, mathematics, and life skills

### Effect of the physical activity intervention on concentration performance

The results of the mixed linear regression models are provided in Table 3, after adjustment for clustering effects of school classes. In Model 1, concentration performance (CP) at post-intervention was used as outcome/dependent variable. After adjusting for confounding variables, this model shows that the physical activity intervention had no statistically significant effect on children’s CP (B = 2.93, 95% CI:-5.01 to 10.86, p = 0.469). Model 1 shows that baseline CP was statistically significant and positively associated with children’s post-intervention CP scores (p<0.001). Children scored 0.8 points higher at post-intervention per additional CP point at baseline. Moreover, Model 1 reveals a significant influence of some cofounding variables. At post-intervention, girls had a 7.05 point higher CP score than boys, and younger children scored 4.65 points higher per additional year of age. Furthermore, the CP increased by 1.27 per score of self-reported physical activity. Better grip strength and higher VO_2_ max were independently and positively associated with higher CP at post-intervention. The mean CP score increased by 1.16 points per kg grip strength and by 0.77 points per ml·kg^-1^·min^-1^ VO_2_ max.

**Table 3:** Effect of intervention condition on the three outcome variables at post-intervention (May 2016), after adjustment for clustering effects of school classes, controlling for baseline levels (February 2015) of selective attention and academic performance, and potential confounders, Port Elizabeth, South Africa.

	Selective attention and academic performance (at post-intervention) as outcome/dependent variables								
	Model 1 Concentration performance (CP)*			Model 2 Error percentage (E%)*			Model 3 End of the year results (EoYR)*		
	B^a^	Estimate^b^ (95% CI)	p-value^c^	B^a^	Estimate^a^ (95% CI)	p-value^c^	B^a^	Estimate^b^ (95% CI)	p-value^c^
	**Baseline levels of selective attention and academic performance**								
**Baseline CP (Model 1), E% (Model 2) and EoYR (Model 3)**	0.80	0.71 to 0.88	<0.001	0.47	0.41 to 0.53	<0.001	0.47	0.41 to 0.54	<0.001
	**Confounders (as assessed at baseline)**								
Gender^d^	7.05	1.98 to 12.11	0.006	-1.86	-3.46 to -0.26	0.023	0.35	0.19 to 0.52	<0.001
Stunted^e^	5.81	-2.23 to 13.85	0.157	0.73	-1.82 to 3.28	0.574	-0.17	-0.42 to 0.08	0.185
Anemic^f^	-1.38	-7.20 to 4.43	0.641	0.57	-1.28 to 2.42	0.544	-0.04	-0.22 to 0.14	0.681
Infected with intestinal protozoa^g^	-1.07	-7.03 to 4.89	0.726	-0.81	-2.70 to 1.08	0.402	-0.04	-0.23 to 0.14	0.641
Infected with soil-transmitted helminths^h^	-5.86	-12.84 to 1.11	0.099	0.05	-1.87 to 1.96	0.960	-0.07	-0.31 to 0.16	0.553
Age in years	-4.65	-8.04 to -1.25	0.007	1.48	0.41 to 2.54	0.007	-0.18	-0.29 to -0.07	<0.001
BMI in kg m^-2^	0.12	-0.69 to 0.94	0.764	-0.13	-0.39 to 0.12	0.313	-0.001	-0.03 to 0.03	0.893
SES^i^	0.57	-0.57 to 1.71	0.325	-0.37	-0.73 to -0.02	0.039	-0.004	-0.04 to 0.03	0.815
Score of self-reported physical activity^j^	1.27	0.25 to 2.32	0.016	-0.34	-0.66 to 0.03	0.031	-0.01	-0.04 to 0.03	0.677
Grip strength in kg	1.16	0.29 to 2.04	0.009	-0.1	-0.37 to 0.18	0.495	0.004	-0.02 to 0.03	0.787
VO_2_ max in ml kg^-1^ min^-1^	0.77	0.12 to 1.42	0.021	-0.16	-0.37 to 0.04	0.114	0.004	-0.02 to 0.02	0.693
**Intervention condition**									
Intervention condition^k^	2.93	-5.01 to 10.86	0.469	-1.05	-0.69 to 2.78	0.237	0.34	0.03 to 0.65	0.032

* In the mixed linear regression models, cases were excluded listwise from the analyses if they had missing data in one or several of the covariates. Thus, all mixed linear regression analyses were based on data of children with complete data records across all variables: n = 521

^a^ B represents the estimate of the beta coefficient

^b^ Adjusted estimates of mean change in the respective outcome from baseline to post-intervention: Unstandardized Beta coefficients, 95% confidence interval, and p-value

^c^ All p-values are calculated using mixed linear regression, adjusting for clustering of school classes.

^d^ Gender, (0 = boys, 1 = girls)

^e^ Stunting is defined as HAZ score ≤-2 (1 = stunted, 0 = normal)

^f^ Anemic is defined as hemoglobin concentration in blood ≤114 g/l, (1 = anemic, 0 = normal)

^g^ Infected with one or two intestinal parasite species (*Cryptosporidium* spp. and/or *Giardia intestinalis*), (1 = infected, 0 = not infected)

^h^ Infected with one or two soil-transmitted helminth species (*Ascaris lumbricoides* and/or *Trichuris trichiura*), (1 = infected, 0 = not infected)

^i^ Socioeconomic status (SES) measured by ownership and housing related questions on a scale from 0 to 9 points (0 = lowest score, 9 = highest score)

^j^ Score of self-reported physical activity for the past 7 days on a scale from 0 to 7 (0 = lowest score, 7 = highest score)

^k^ School children from the intervention group took part in a 20-week physical activity intervention program, as described in Table 3 and Fig 2 (1 = intervention group, 0 = control group)

### Effect of the physical activity intervention on error percentage

Model 2 of the mixed linear regression analyses (see Table 3) suggests that after having adjusted for clustering effects of school classes, baseline levels of E% and potential confounders, the physical activity intervention had no significant effect on E% at post-intervention (B = -1.05, 95% CI:-0.69 to 2.78, p = 0.237). The mixed linear regression model further shows that baseline E% was significantly and positively associated with E% at post-intervention (p<0.001). Children’s E% was 0.47% lower at post-intervention per additional percent at baseline. For every year younger a child was, children made 1.48% fewer errors. Whereas girls made 1.86% fewer errors than boys, children with higher SES and self-reported physical activity scores made fewer errors. The mean E% decreased by 0.37 percent per additional SES point and decreased by 0.34 percent per additional day of self-reported physical activity.

### Effect of the physical activity intervention on EoYR academic results

Most importantly, Model 3 of the mixed linear regression analyses (see Table 3) shows that after accounting for clustering effects of school classes and controlling for baseline levels of academic performance as well as confounding factors, the physical activity intervention significantly predicted the EoYR at post-intervention (B = 0.34, 95% CI: 0.03 to 0.65, p = 0.032). However, as can be seen on a descriptive level (Table 2), this intervention effect was primarily based on EoYR remaining stable in the IG, whereas scores decreased in the CG. Additionally, the mixed linear regression shows that baseline EoYR were statistically significantly and positively associated with the post-intervention EoYR (p<0.001). Children’s school grades were 0.47 grades higher at post-intervention per additional grade at baseline. Girls had 0.35 grades higher EoYR than boys. Finally, for every year younger a child was, children had 0.18 grades higher EoYR at post-intervention.

## Discussion

The most important finding of the present study is that a 20-week physical activity intervention had a positive effect on children’s EoYR. Indeed, the academic performance remained stable in children in the IG, whereas a decrease by half a grade was observed in the CG. Yet, no effects of the physical activity intervention were found on selective attention.

Our results are in line with previous studies showing maintenance and/or a smaller decline of academic performance in children participating in a physical activity intervention, compared to those experiencing no change in physical activity levels [39, 40]. Our findings thus indicate that physical activity promotion may be a strategy to maintain academic performance with increasing demands. Other studies found enhancing effects within this domain [41]. For example, Hollar et al. [42] reported improved reading and mathematics skills after a 1-year physical activity intervention, in combination with a nutrition intervention in elementary school children from low-income families. Similarly, Chaya et al. [43] found that a 3-month physical activity intervention, including yoga and physical education lessons, had a positive impact on cognitive performance (arithmetic, coding, and vocabulary) in socioeconomically disadvantaged Indian school children. Szabo-Reed et al. [44] reported that moderate to vigorous physical activity lessons were significantly associated with more on-task time behavior.

In the present study, a possible explanation for the decreased academic performance in the CG could be the transition of language of instruction, also referred to as the “fourth-grade slump” [45]. The baseline results refer to the final grades of grade three learners and the post-intervention results refer to the final grades from grade four. In South Africa, most schools offer mother-tongue instruction for the first three grades of school, while the transition to English as the language of instruction occurs in grade four [46]. Children undergo a shift from “learning to read” to “reading to learn”, in conjunction with a change in the language of instruction. Cummins [47] argues that only after the mastery of the first language a child will have the necessary skills to transition to a second language. Thus, our findings are in line with previous studies [45, 48], which show that academic performance is decreasing in South African school children progressing from grade three to grade four. Hence, one interpretation of the present study’s results could be that increasing in-school physical activity levels in third grade schooling holds promise to counteract a negative tendency of academic performance as transitioning to English language instruction.

Our findings further suggest that self-perceived physical activity, cardiorespiratory fitness, and grip strength were independently associated with selective attention. The first finding accorded well with previous studies showing that higher physical fitness levels are associated with better cognitive performance. For instance, our results are similar to a study conducted by London et al. [49], in which overall physical fitness predicted academic performance. In a recent review, Donnelly et al. [41] stated that increased cardiovascular fitness and physical activity has been associated with improved cognitive function, brain structure and function, and academic performance. Although cardiorespiratory fitness and physical activity are not identical concepts [50], physical activity can be considered as a proxy measure for physical fitness, particularly as fitness is a potential outcome of regular physical activity participation [51]. Hence, our findings corresponds with earlier studies showing that a positive relationship exists between physical activity and cognitive performance and academic performance [40]. However, as attention remained unchanged, this cognitive domain cannot explain why a decline in academic performance was observed in the CG only. Although children with higher physical fitness levels and higher self-reported physical activity seem to have an advantage in paying attention compared to their less fit peers, this does not necessarily guarantee the maintenance of academic performance. Evidence from longitudinal studies suggests that physical activity has an indirect effect on academic performance through a pathway of executive function (i.e., top-down control of behavior, especially in non-routine situations) [41]. The direct effect of physical activity on this higher-order cognitive function is well documented [52] and has been attributed to morphological changes (i.e., angiogenesis, neurogenesis, and synaptogenesis) in brain regions that are important for learning [53]. Another pathway that has been discussed as a potential mechanism underlying executive function benefits is the exercise-induced psychological stimulation that contributes to the improvement of self-control ability, which may impact on academic performance [54]. Although speculative, it seems that changes in higher-order cognitive functions rather than attention may have contributed to the maintenance of academic performance in the IG. This suggests that physical activity, and therefore physical fitness, may positively affect important brain areas that stimulate cognition and as a result give fit children an advantage compared with their less fit peers [39].

In contrast, Spitzer et al. [55] and Adsiz et al. [56] found that a physical activity program has the potential to enhance selective attention. Hence, study results seem to vary considerably, most likely due to differences in the mode of assessment of selective attention, the setting in which the study took place, or the nature and intensity of the physical activity intervention. In the present study, the intervention might not have resulted in a positive effect because the intervention period was relatively short (total of 20 weeks) and because of the context in which the intervention took place (e.g., large class sizes, heterogeneous student population in terms of age and academic performance). These factors have complicated the implementation of the intervention and may have hindered the detection of subtle effects.

To our knowledge no study has examined the effect of a physical activity program on socioeconomically underprivileged children’s selective attention, only acute effects have been reported [57]. Given these findings, new research is needed to deepen the understanding of whether and how a physical activity might be associated with SES.

Our findings further show that older children have significantly lower scores for selective attention and academic performance. This may be explained by the fact that disadvantaged communities do not have the financial means to promote children with special needs or learning disabilities [58]. Children suffering from attention deficit hyperactivity disorder (ADHD), fetal alcohol syndrome, reading difficulties, or neglect, might not get the required academic support and, subsequently, they might not be able to keep up with their peers. Learners with inadequate grades are retained up to three years until they become too old and automatically progress to the next grade [58, 59]. Moreover, girls seemed to achieve better academic results compared to boys and achieve higher scores for selective attention. This is in line with a meta-analysis by Voyer and Voyer [60], in which a consistent female advantage with regards to school grades was found for all subject content areas.

Our study has several limitations. First, academic performance was operationalized by the average end of the year grade (achieved at the end of grade three and four), which corresponds to the summary of four subjects (i.e., mathematics, home language, additional language, and life skills). While the objectivity of school grades can be questioned as a reliable outcome in empirical research (e.g., due to attributions or stereotypes of the teachers and/or different standards between classes/schools) [61], this measure has a high ecological validity because sufficiently high grades are needed for academic promotion, and the present study showed that selective attention and the academic performance scores were moderately correlated (r >0.30).

Second, allocation to the intervention and control condition was done school-wise. We are aware that random allocation at class level is the ‘gold’ standard, but this was difficult to achieve in the present study. For instance, one component of the physical activity intervention was the creation of a “physical activity friendly” school environment. Thus, changes in the infrastructure were performed, which cannot be isolated for learners from specific classes. Nevertheless, we considered school class as random factor in our multivariate regression analyses to account for the variation in academic performance and intervention implementation between schools and classes.

Third, we used an indirect measurement of VO_2_ max to assess aerobic fitness and children might not have performed to their best abilities due to lack of motivation. However, this standardized test was chosen because it is well-suited for a resource-constrained setting due to its ease of application [30].

Fourth, physical activity was assessed with a single self-reported item about children’s physical activity levels. While it can be questioned whether children are able to accurately respond to this item, previous data from the present study have shown that children with higher physical activity levels indeed report higher health-related quality of life [62], have lower blood pressure scores, and are less likely to be overweight or obese [63].

Fifth, the physical activity intervention module was well perceived by the children and teachers alike. Yet, the level of the teacher’s compliance and adherence toward a high intervention quality varied considerably. For instance, some of the teachers in the intervention schools did not have a high motivation to be physically active. Furthermore, the length of the intervention was relatively short. Hence, it may be that a longer intervention period is needed to positively impact selective attention among primary school children.

Sixth, as acknowledged previously, the present study took place in disadvantaged communities (quintile three schools). Consequently, variation in SES was limited, which might have resulted in an underestimation of SES as a predictor of selective attention and academic performance.

Seventh, on a descriptive level, baseline differences were apparent between the IG and CG in academic performance, although these schools were similar in size and student population. Such baseline differences pose a challenge with respect to data analyses, as the impact of the intervention could be interpreted as a regression to the mean. In other words, since learners of the CG had higher scores at baseline, there was also more scope for a decrease compared to peers from the IG.

These shortcomings should be addressed in future research by either using study designs that allow a class-wise group assignment or by controlling more systematically for academic performance when schools are selected to ensure that no between-school differences exist in children’s EoYR prior to the beginning of the intervention program.

## Conclusion

Participation in a 20-week physical activity intervention implemented in disadvantaged schools in Port Elizabeth, South Africa, was positively associated with children’s academic performance. Our findings suggest that such a physical activity intervention has the potential to counteract decrease in academic performance. Hence, reintegration of physical education into the curriculum might have beneficial effects for children’s academic performance. Yet, this conclusion needs to be interpreted with caution because the intervention period was relatively short, compliance was uneven, and there were differences in academic performance between the IG and CG at baseline. Future research is needed in disadvantaged schools with a physical activity intervention being carried out over an extended period of time and allocation procedures used that minimize the risk of baseline group differences.

## Supporting information

S1 File Study protocol submitted to the EKNZ (2014). (PDF)

S1 Table Consort checklist 2010. (DOC)
