# Supplementary material for: Effect of a 20-week physical activity intervention on selective attention and academic performance in children living in disadvantaged neighborhoods: A cluster randomized control trial
Source: PLoS One. 2018 Nov 8;13(11):e0206908. doi: 10.1371/journal.pone.0206908 (PMC6224098; doi:10.1371/journal.pone.0206908)
Supplement: S1 File — (PDF) [file pone.0206908.s001.pdf]

Version 1, 8 May 2014

Study protocol

Impact of disease burden and setting-specific interventions on schoolchildren's physical fitness and psychosocial health in Port Elizabeth, South Africa

---

Table of content

1. Title 3

1.1. List of investigators 3
1.2. List of collaborators 4

2. Project description 5

2.1. Background 5
2.2. Goal and specific objectives 6
2.3. Study area, framework and participants 6
2.4. Project hypothesis 6

3. Experimental design 7

3.1. Study design 7
3.2. Sample size calculation 9

4. Study population 10

4.1. Enrolment and written informed consent 10
4.2. Inclusion and exclusion criteria 11

5. Assessment methods 11
6. Statistical analysis 16

6.1. Data collection and management 16
6.2. Methods for data analysis 17

7. Ethical considerations 17

7.1. Ethical committee review 17
7.2. Duties of investigators 17
7.3. Confidentiality 17

7.4. Feed-back at the end of the study 18
7.5. Treatment 18
7.6. Risks and benefits of the study 18

8. References 19

Prof. Dr. med Christoph Hatz | Prof. Dr. Uwe Pühse | Prof. Dr. Jürg Utzinger

1. Title

Impact of disease burden and setting-specific interventions on schoolchildren's physical fitness and psychosocial health in Port Elizabeth, South Africa

1.1. List of investigators

Prof. Dr. Uwe Pühse, Principal-investigator
Departement für Sport, Bewegung und Gesundheit (DSBG)
Universität Basel
CH-4052 Basel, Switzerland
Tel.: +41 61 377-8780/84
E-mail: uwe.puehse@unibas.ch

Prof. Dr. Jürg Utzinger, Co-investigator
Department of Epidemiology and Public Health
Swiss Tropical and Public Health Institute
CH-4002 Basel, Switzerland
Tel.: +41 61 284-8129
E-mail: juerg.utzinger@unibas.ch

Prof. Dr. Rosa du Randt, Co-investigator
Department of Human Movement Science
Summerstrand Campus (South)
Nelson Mandela Metropolitan University
Port Elizabeth 6031, South Africa
Tel.: +27 41 504-2499/7
E-mail: rosa.durandt@nmmu.ac.za

Prof. Dr. Cheryl Walter, Co-investigator
Department of Human Movement Science
Summerstrand Campus (South)
Nelson Mandela Metropolitan University
Port Elizabeth 6031, South Africa
Tel.: +27 41 504-2628
E-mail: cheryl.walter@nmmu.ac.za

1.2. List of collaborators

Bruce Peter Damons, research team adviser
Principal of the Sapphire Road Primary School
211 Mountview Drive, Malabar, Port Elizabeth, 6020, South Africa
Tel.: +27 41 483-1878
E-mail: bpdamons@vodamail.co.za

PD Dr. Markus Gerber
Departement für Sport, Bewegung und Gesundheit (DSBG)
Universität Basel
CH-4052 Basel, Switzerland
Tel.: +41 61 377-8783
E-mail: markus.gerber@unibas.ch

Dr. Harald Seelig
Departement für Sport, Bewegung und Gesundheit (DSBG)
Universität Basel
CH-4052 Basel, Switzerland
Tel.: +41 61 377-8786
E-mail: harald.seelig@unibas.ch

Dr. Peter Steinmann
Department of Epidemiology and Public Health
Swiss Tropical and Public Health Institute
CH-4002 Basel, Switzerland
Tel.: +41 61 284-8229
E-mail: peter.steinmann@unibas.ch

Dr. Peiling Yap
Department of Epidemiology and Public Health
Swiss Tropical and Public Health Institute
CH-4002 Basel, Switzerland
Tel.: +41 61 284-8603
E-mail: peiling.yap@unibas.ch

Ivan Müller
Department of Epidemiology and Public Health
Universität Basel
CH-4052 Basel, Switzerland
Tel.: +41 41 535-1292
E-mail: ivan.mueller@unibas.ch

2. Project description

2.1. Background

Key findings from the Global Burden of Disease study 2010 (GBD 2010), published in a collection of papers in the Lancet in December 2012, reveal the current global health status. Compared to 1990, we are living longer but with poorer health. Encouragingly, a 60% drop in under-5 mortality has occurred since 1990. Globally, HIV/AIDS is now the single largest cause of death in adult women (14.4%), whereas road traffic accidents now match HIV/AIDS (10.7%) as the second leading cause of death in men. Physical inactivity and inappropriate diet (most importantly low fruit intake and excess salt consumption) have emerged as new leading risk factors, accounting for 10% of the global burden of disease, as expressed in disability-adjusted life years (DALYs). Overweight has replaced under-nutrition as a risk factor for the first time in history [1-3]. Chronic disease is replacing premature mortality everywhere in the world, except in Africa, where a range of infectious diseases is still rampant and costing lives at a young age. It must be noted, however, that these global averages hide important information, and hence, what is happening locally, nationally and regionally is more crucial for research, as such evidence will provide the basis for intervention and mitigation of risk factors to improve health and enhance general wellbeing.

A recent research project, pursued by Prof. Cheryl Walter and her team from the Nelson Mandela Metropolitan University in South Africa, investigating the in-school physical activity patterns of primary schoolchildren at disadvantaged schools in South Africa confirmed that levels of physical activity among school-aged children are insufficient in South Africa [4]. In turn, physical inactivity leads to poor health outcomes in children and adolescents. Further research by Prof. Cheryl Walter and Prof. Rosa du Randt on the physical activity and health status of black South African women has revealed similar results [5]. Hence, there is a need to promote physical activity among young people in South Africa. This is especially the case among disadvantaged South African schools and communities, which are not conducive for the promotion of physical activity due to inadequate sport and recreation facilities, a lack of qualified teachers and an irregular physical education schedule. It is hypothesized that due to decreased physical activity, obesity in school-aged children is further exacerbated. Surveys and further studies have revealed that the South African population has moved towards a disease profile similar to Western countries, with increasing proportions of deaths attributed to chronic diseases of lifestyle [6]. In 2010, Kimani-Murage *et al.* [7] reported that in a low-income South African setting, the co-prevalence of early stunting and adolescent obesity (in girls) is a result of increasing levels of physical inactivity. This observation was particularly prevalent among black women, who suffered from the highest levels of physical inactivity [8]. Consequently, individuals suffering from obesity are at the greatest risk of developing chronic diseases of lifestyle. Despite on-going research and a growing body of knowledge on physical activity in South Africa, there is still a dearth of knowledge regarding the determinants and constraints of participation in physical activity.

Additionally, infectious diseases that are intimately connected to poverty may also occur in disadvantaged South African schools [9]. Consequently, these infections might have a negative impact on cognitive abilities and, hence, on children's school performance [10]. This potential dual burden of diseases (i.e. non-communicable chronic conditions and infectious diseases) puts these children at an especially high risk of compromised health that may hamper growth and development. Today, the World Health Organization (WHO) finds itself needing to deal with the new pandemic of obesity and its accompanying non-communicable diseases since childhood malnutrition has disappeared in many places. Concurrently, tuberculosis (TB) and malaria rates are still escalating, and there is a pandemic of HIV/AIDS. This has created a dual burden of disease that threatens to overwhelm the health services of many resource-poor countries. Therefore, in-depth epidemiological studies of this potential

combined disease burden in an impoverished region of South Africa can provide unique opportunities for research on its impact on children's physical fitness, cognitive performance and psychosocial health and eventually, a concerted response in the form of research-based intervention studies.

2.2. Goal and specific objectives

The overarching goal of this project is to assess the burden and distribution of communicable diseases and non-communicable chronic conditions among school-aged children in selected schools near Port Elizabeth, South Africa, and to assess their impact on children's physical fitness, cognitive performance and psychosocial health. This information will allow the improvement of overall child health by designing and introducing key health interventions and rendering the school infrastructure more amenable for physical activity.

This goal will be achieved by pursuing the following objectives:

(i) To conduct a rapid appraisal in approximately 50 schools of the Port Elizabeth area (20 children per school) in order to determine the extent of non-communicable chronic conditions (e.g. type 2 diabetes and obesity) and communicable diseases (e.g. helminth infections and malaria).

(ii) To design and implement a cluster randomized trial assessing the effect of setting-specific interventions (e.g. lifestyle interventions and deworming) on improving children's health and wellbeing. Thirty schools (30 children per school) will be selected. Assessment of anthropometric indicators (e.g. height, weight and body composition), physical fitness levels cognitive performance and psychosocial health will be conducted. These findings will be further correlated with the prevalence of communicable and non-communicable diseases in the study population.

2.3. Study area, framework and participants

The proposed study will be conducted in historically black primary schools in the Port Elizabeth township of South Africa. These schools and communities are detrimentally affected by extreme poverty and high unemployment due to past colonial and apartheid policies as well as the current public health and economic challenges faced by the country [11]. The school principal of Sapphire Road Primary School, Bruce P. Damons, who has extensive knowledge and experience in school and community health, has accepted the role of the research team advisor. The first stage of the study will be conducted among 1,000 school-aged children (age range 9-12 years) from 50 schools (20 students per school). Under the second stage of the study, 900 students (age range 9-12 years) will be recruited from 30 schools (30 students per school).

2.4. Project hypothesis

An integrated approach consisting of assessments of diseases status and development of health interventions can decrease the incidence of communicable diseases and non-communicable conditions, and therefore improve children's physical health, measured by their fitness, cognitive performance and psychosocial health.

More specifically, we hypothesize that:

(i) A dual burden of communicable diseases and non-communicable chronic conditions among school-aged children in selected schools near Port Elizabeth, South Africa, has a negative influence on the children's physical fitness, cognitive performance and psychosocial health.

(ii) Specific health interventions (e.g. lifestyle interventions and deworming) can improve children's health and wellbeing.

(iii) Combining health interventions designed based on the result of the initial survey with rendering the school infrastructure more amenable for physical activity will have a positive impact on children's health and wellbeing.

3. Experimental design

3.1. Study design

This 3-year project will be conducted between April 2014 and March 2017 in two main stages. The first stage will involve a **rapid cross-sectional appraisal**, which will take approximately 6 months. It will be conducted among 1,000 school-aged children (age range 9-12 years) from 50 schools (20 students per school), using a suite of standardised, quality-controlled techniques in order to determine the spectrum and magnitude of communicable diseases and non-communicable chronic conditions among study participants. A clinical examination, which can allow us to identify approximately 70% of the health problems of each child, followed by anthropometric measurements and a quick parasitological screen, will be performed for each child. This will allow us to gather health information on the local schoolchildren population and identify schools for the intervention trial in the second stage of this project.

Based on the rapid appraisal survey, the development of setting-specific interventions will be pre-tested and further implemented in a **cluster randomized controlled trial,** which will last for approximately 1.5 years (Figure 1). We will select approximately 30 disadvantaged schools, out of the 50 schools included in the rapid appraisal, whereby 15 intervention and 15 control schools will be randomly assigned. Disease status, physical fitness, cognitive performance and psychosocial health will be monitored in 30 students per school at baseline, mid-point of the intervention and again upon completion. Finally, consolidation and communication of results to the scientific community and relevant authorities will be performed at the end of the project (Table 1).

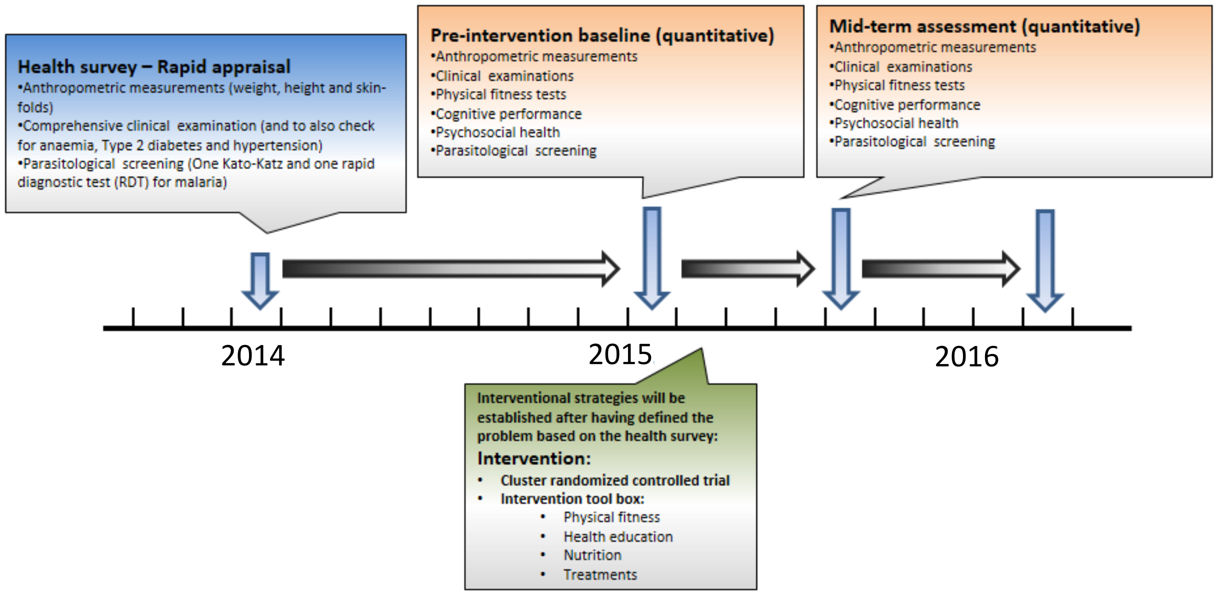

Figure 1. The figure displays the conceptual framework and proposed timing of the study.

Cluster randomized controlled trial:

Implementation of interventions for the cluster randomized trial

The following intervention toolbox is proposed but the specific combination of interventions to be used will be governed by the key findings from the initial cross-sectional rapid appraisal.

Intervention tool box:

(I) The PasSPORT to Health Project initiated by Prof. Cheryl Walter in 2010, can be replicated and adapted in the schools from Port Elizabeth. The primary aim of the PasSPORT to Health project is to enable human movement science (HMS) students from the Nelson Mandela Metropolitan University (NMMU) to learn and promote physical activity and school sport at disadvantaged schools in Nelson Mandela Bay. Interventions tailored to the needs of schools include the creation of physical activity friendly school environment. This is jointly developed by students, teachers and parents, and will bolster a sense of community ownership and a sense of empowerment that the community is taking steps to improve overall health.

(II) Physical fitness programs [12, 13] targeting non-communicable conditions: regular physical activity opportunities incorporated into the main school curriculum should change participant's personal health condition. Leadership camps can be conducted among students to facilitate sports coaching.

(III) Health education [14]: This will help increase the awareness for health conditions among the students and educate them on treatment and prevention methods. Counselling could be done on a per student basis. Self-control training (e.g. regular tasks to improve posture, use of non-preferred hand, verbal self-regulation including avoiding curse words, speaking in complete sentences), which has been shown to positively impact young people's health-behaviours, well-being and academic performances in previous studies could also be included [15-18]. Importantly, regular exercise has also been shown to positively affect self-control and to improve behavioral outcomes [19].

(IV) Nutritional intervention: Depending on the disease status of the population, nutritional intervention could be introduced in the form of multi-micronutrient supplementation (for undernutrition and infectious diseases) and/or dietary improvements in school and home meals (for non-communicable diseases). Nutritional counseling could also be done on a per student basis.

(V) Treatments of communicable diseases: This could be anthelminthics for intestinal helminth infections (albendazole: single dose of 400 mg; praziquantel: 40 mg/kg [20]) and artemisinin-based combination therapy (ACT) for malaria.

Year	2014				2015				2016				2017	
**Activity** Quarter	1	2	3	4	1	2	3	4	1	2	3	4	1	2
**A1: Preparatory work**														
Protocol writing, ethical approval, trial registration		■	■											
**A2: Establishing study sites in South Africa**														
Kick-off workshop			■											
**A3: Rapid formative appraisal**														
Clinical examination, anthropometric measurements, parasitological examination, physical fitness, cognitive performance and psychosocial health questionnaires				■	■									
**A4: Cluster randomized trial**														
Development and pre-testing of interventions; random assignment of intervention and control schools					■									
Baseline assessment of the same parameters used at appraisal						■								
Start of interventions						■	■	■	■	■	■			
Mid-term evaluation of the same parameters used at baseline								■						
Post-trial assessment of the same parameters assessed at baseline												■		
**Consolidation, writing-up, dissemination**										■	■	■	■	■

Table 1. Approximate time schedule of the proposed 3-year project.

3.2. Sample size calculation

The sample size calculation of the cross-sectional appraisal of this study is based on equation 1 [21], which is illustrated below, where the design effect is shown and equation 2 (cluster formula). The design effect is an adjustment used in studies, such as cluster randomized trials, to allow for correlations among clusters of observations [22]. The smaller the value of the design effect, the better the overall reliability of the sample estimate. In general, for a well-designed study, the design effect is

usual slightly above one. Under the assumption that a normal intra-class-correlation (ICC) is 0.2 and we have planned to examine 20 schoolchildren per school, we get in our study a design effect of 4.8.

$$\boldsymbol{D_{eff} = 1 + (m - 1) * ICC} \qquad \textbf{Equation 1}$$

Legend:

$D_{eff} = Design\ effect$

$m = Number\ of\ investigated\ schoolchildren\ per\ school$

$ICC = Intra - class - correlation\ (normally\ between\ 0.1 - 0.2)$

With an expected disease prevalence of p = 0.15 (q = 0.85), the result out of the following cluster formula (equation 2) is 49 clusters (schools). Because of the potential number of schoolchildren lost to follow-up, we plan to carry out the cross-sectional appraisal of the study with 50 schools (clusters).

$$\boldsymbol{clusters = \frac{p * q * D_{eff}}{se^2 * b}} \qquad \textbf{Equation 2}$$

Legend:

$b\ = Number\ of\ examined\ children\ per\ school\ (= 20)$

$empirical\ standard\ deviation\ (se) = 0.025\ (i.e. 95\%\ CI\ ^{+}/- 5\% - points)$

In order to have sufficient statistical power in interpreting the final results, we are planning to include into the following cluster randomized controlled trial 30 schools (15 schools with interventions and 15 control schools) with each 30 schoolchildren per school.

4. Study population

4.1. Enrolment and written informed consent

School authorities will first be contacted. Subsequently, school directors and teachers of the selected schools will be informed about the objectives, procedures and potential risks and benefits. Thereafter, schoolchildren, parents or legal guardians of students will be informed and all children will be encouraged to participate in the study. Before launching the cross-sectional baseline survey to outline the extent of communicable diseases and non-communicable chronic conditions and providing subsequent treatments, a patient information sheet that is designated for the schoolchildren and parents/guardians will be given to all participants explaining the objectives, procedures and potential risks and benefits of the study. The names and contact address of the main investigators on site will be provided, so that they can be contacted anytime. Oral assent for each participating schoolchild will be sought and individual written informed consent will be obtained from parents/guardians. For illiterate parents, the information sheet will be read aloud and, if need be, an oral translation of the information sheet into the local languages will be provided. It will be emphasized that participation is voluntary and children can withdraw from the study at any time without consequences and further obligation.

4.2. Inclusion and exclusion criteria

Schoolchildren will be invited to participate in the study

– if they do present any of the following inclusion criteria:

(1) willing to participate in the study;
(2) be in possession of a written informed consent by a parent/guardian on behalf of the child;
(3) not participating in other studies; and
(4) being a primary schoolchild aged 9-12 years, male or female.

– and if they do not present any of the following exclusion criteria:

(1) not having a written informed consent or no parental/legal guardian's permission to participate;
(2) suffer from medical conditions which prevent participation in the study as determined by qualified medical personnel; and
(3) attending other clinical trials during the study period.

5. Assessment methods

For both the cross-sectional rapid appraisal and the cluster randomized controlled trial, a combination of the following procedures will be selected and conducted, based on the local disease setting, by well-trained staff adhering to standardised, quality-controlled protocols:

Parasitological examinations

(I) Single stool sample will be collected from each child and analyzed on the same day. Stool sample (at least 15 *g*) will first be visually examined for the presence of *Taenia* spp. proglottids as well as signs of blood, mucus and diarrhoea. Second, duplicate 41.7 *mg* Kato-Katz thick smears will be prepared from each stool sample [23]. Slides will be allowed to clear for 30-45 min. Slides will be examined under a microscope by experienced laboratory technicians and the number of helminth eggs counted and recorded for each species separately. Helminth egg counts will be multiplied by a factor 24 to obtain a proxy for infection intensity, as expressed by the number of eggs per 1 *g* of stool (EPG) [24, 25]. Possible species to be detected include the three main species of soil-transmitted helminths (i.e. *Ascaris lumbricoides*, hookworm and *Trichuris trichiura*), *Fasciola hepatica* and *Schistosoma mansoni*. Third, approximately 10 *g* of stool will be used to perform the Baermann test [26, 27]. In this test, the stool sample is placed on medical gauze in a glass funnel and covered with tap water. The whole apparatus is exposed to artificial light directed at the bottom of the sample. After 2 hours, the lowest portion of the liquid (50 ml) is collected, centrifuged (500 *g* for 2 min) and the sediment is subjected to microscopic examination (40x magnification) for the larvae of *Strongyloides stercoralis*. Fourth, 1-2 *g* of stool will be placed on an agar plate for evaluation according to the Koga method [28]. In this method, the stool sample is placed in the middle of a freshly prepared agar plate (agar media: 1.5% agar, 0.5% meat extract, 1.0% peptone, 0.5% sodium chloride) and the closed Petri dish is incubated in a humid chamber for two days at ambient temperature before being rinsed with 10 ml sodium acetate acetic acid-formalin (SAF). The eluent is centrifuged at 500 *g* for 1 min and the sediment examined under a microscope (40x magnification) for the larvae of hookworm and *S. stercoralis* [24, 29]. Finally, for each

individual, a weighed amount of stool (between 1 and 2 *g*) will be thoroughly mixed with 10 ml of SAF solution and semi-quantitatively examined in reference laboratories for helminths and intestinal protozoa, using an ether-concentration method [30]. The steps in brief include first, re-suspension of SAF-fixed stool samples and straining through gauze into a centrifuge tube. Second, centrifuging the tube for 1 min at 500 *g*. Third, decanting the supernatant, if the final sediment contained more than 1 ml, the first two steps are repeated or the sediment is re-suspended in 0.9% NaCl and part of the suspension removed. Fourth, adding 7 ml of 0.9% NaCl plus 2–3 ml diethyl ether to the remaining sediment, then closing the tube with a rubber stopper, shaking for approximately 30 sec and centrifuging for 5 min at 500 *g*. Finally, from the four layers formed, the three top layers were discarded and the resulting sediment is examined microscopically for intestinal helminthes and protozoa.

(II) For the detection of *Cryptosporidium* spp. and *Giardia intestinalis*, a Crypto-Giardia Duo-Strip® rapid diagnostic test (RDT) will be performed on a stool sample which has been diluted with a commercialized buffer.

(III) For additional examinations on the stool sample (e.g. bacterial and viral multiplex PCR, bacterial culturing), an aliquot of the stool sample will be prepared by adding 500 *mg* of the stool in 2 ml of 96% ethanol. This aliquot will be stored in a refrigerator pending analysis.

(IV) Single urine sample from each child will be collected and analysed visually for macro-haematuria and tested with reagent strips to detect blood in urine as a proxy for *Schistosoma haematobium*. The urine filtration method is used to detect *S. haematobium* eggs. In brief, samples will be vigorously shaken and 10 ml filtered through a 13 ml nylon filter with a syringe. Filters will be put on microscope slides, a drop of Lugol added, and *S. haematobium* eggs counted under a microscope. Infection intensity will be expressed as the number of eggs/10 ml of urine. Finally, the point-of-care circulating cathodic antigen (POC-CCA) test will be used to detect the presence of *S. mansoni* infections [31].

(V) For the detection of malaria, an RDT involving a drop of whole blood from a finger prick will be used [32].

(VI) For quality control, a random sample of 10% of all Kato-Katz and urine filtration slides will be re-examined by a senior technician. In case of discordant results, the slides will be read a third time and results were discussed among the technicians until agreement has been reached. The collection of stool and urine samples from each participant and the subsequent diagnostic work-up with a suite of standardised, quality-controlled methods is mandatory to have a reasonably sensitive diagnostic accuracy [24, 26].

Clinical examinations

(I) Clinical examination of children includes detailed history taking and physical examination to assess for presence and complications of infections. Features of patient history should focus on fevers, constitutional symptoms, abdominal pain and change in bowel movements. Additionally, corroborative history from parents should be included where possible, and focus on developmental cognitive and physical milestones. Physical examination is directed towards evidence of anaemia (e.g. conjunctival pallor), detailed abdominal examination (e.g. tenderness, hepatomegaly, splenomegaly), and evidence of pulmonary hypertension (jugulovenous pressure, cardiac auscultation). In addition, a physical examination for specific micronutrient deficiencies and muscle mass at targeted sites (e.g. deltoid, vastus medialis) will also be conducted.

(II) For the detection of anaemia, the haemoglobin concentration will be measured once (to the nearest 0.1 *g*/l) with a HemoCue® Hb 301 system. A fresh set of alcohol swab, safety lancet and microcuvette will be used for each child. After swabbing the fingertip with alcohol, the field worker will prick it with a safety lancet and squeeze gently for two drops of blood. The first drop will be wiped away with the alcohol swab and the second drop will be taken up by

the microcuvette and read by the machine. (Note: since there will be other tests, which involve the use of whole blood from a finger prick, we will ensure a highly organized procedure so that each child is only pricked once.)

(III) For the detection of hypertension, blood pressure of each child will be taken once with the Omron® digital blood pressure monitor while the child is seated. The cuffs will be wrapped around the left arm such that one finger could fit between the cuff and the arm. The bottom of the cuff should be about 4.0 cm above the elbow and the palm should be facing up while the blood pressure is being taken.

(IV) For the detection of type 2 diabetes, blood glucose level will be measured once using the Accu-Check® blood glucose monitoring system. A fresh set of alcohol swab, safety lancet and test strip will be used for each child. After swabbing the fingertip with alcohol, the field worker will prick it with a safety lancet and squeeze gently for two drops of blood. The first drop will be wiped away with the alcohol swab and the second drop will be taken up by the test strip and read by the machine.

Anthropometric measurements

(I) Each child will be asked to take off his/her shoes and sweater before standing on the digital weighing scale. Body weight will be measured (to the nearest 0.1 kg) twice and averaged.

(II) With the shoes off, each child will stand against a stadiometer with his/her back erect and shoulders relaxed. Body height will be taken (to the nearest 0.5 cm) twice and averaged.

(III) Body mass index (BMI) and 2 Z-scores will be calculated. BMI = weight (kg) / (standing height [meters (m)]^2); BMI-for-age (BMIZ: an indicator for weight-for-height proportion; WHO growth charts [33]); and height-for-age (HAZ: an indicator for chronic nutritional status; WHO growth charts).

(IV) The thickness of the skinfold will be measured at 2 sites, namely triceps and subscapular [34, 35]. Before the measurement begins, the field worker will show the Holtain skinfold caliper to the child and clamp it normally on the child's finger to show that the process will not hurt. During the measurement, the child will stand with arms and shoulders relaxed. With the thumb and forefinger, the field worker will gently pinch the skin (a vertical skinfold) slightly above the middle of the back of the arm (triceps) and clip the caliper (mouth of caliper is perpendicular to skinfold). After counting for 4 sec, the reading should stabilize and be recorded. The field worker will release the pinch but let the fingers stay in the same position on the arm and repeat the measurements 2 additional times. The 3 values obtained should be no more than ±5% different from each other. If this is not the case, the measurements will be repeated. The final reading will be an average of the 3 values. The same procedure applies for the subscapular site directly underneath the shoulder blade.

Physical fitness tests

Studies in South Africa have used the Eurofit fitness testing battery [36]. For the purpose of this project, specific tests from the Eurofit fitness testing battery will be conducted.

(I) Monitoring physical activity is essential for understanding the determinants of physical activity behaviour in schoolchildren of the selected schools [37, 38]. The Actical accelerometer-system (Koninklijke Philips Electronics, Amsterdam, Netherlands) will allow physical activity to be monitored for days or weeks in a simple and reliable way by means of measuring the proper acceleration. An accelerometer will facilitate the collection of valuable physical activity and energy expenditure data and allow us to identify and characterize behavioral patterns. We have planned to use the accelerometer for 7 consecutive days and periodically throughout the year to account for seasonal changes.

(II) Spirometry, a measurement of breath, is an important tool to assess conditions such as asthma or other respiratory lung diseases, e.g. pulmonary fibrosis - scarring of the lung, both symptoms

of breathlessness [39, 40]. The amount (volume) or speed (flow) of air that can be inhaled and exhaled are common measurements for lung function. The study participant is asked to take the deepest breath he is able to, exhale into the spirometer (Minispir Light, Dieckhoff & Ratschow Praxisdienst GmbH & Co. KG, Longuich, Germany) as hard as possible and for as long as possible, preferably for at least 6 sec. The spirometer will display the following spirograms. A volume-time curve, showing volume (liters) along the Y-axis and time (sec) along the X-axis and a flow-volume loop, which graphically depicts the rate of airflow on the Y-axis and the total volume inspired or expired on the X-axis, with the possibility of before-and-after comparison.

(III) The children's endurance will be measured with the 20-m shuttle run test [41]. The 20-m flat running course will be measured with a measuring tape and marked with cones. In particular, 5 running lanes will be created. Before the start of the test, all the children will be told to voice out any body discomfort and anyone who feels sick or not comfortable will not take part in the test. The pre-recorded sound signals will be played to the children and they will get to do a trial run of 2 intervals (40 m). Once they are familiar with the test procedures, they will be asked to run, in groups of five or ten, back and forth on the 20 m flat course by following the pace of sound signals. Starting with a running speed of 8.5 km/h, the frequency of the signal increases gradually such that every minute, the pace increases by 0.5 km/h. When a child fails to follow the pace in two consecutive intervals, he/she will be asked to stop and the stage and the distance completed fully will be recorded. The age of the participating child and the speed at which the child stopped running will be converted into the maximum volume of oxygen that can be utilized within 1 min during exhaustive exercise (VO_2 max).

(IV) Lower body strength will be estimated with the standing broad jump test. Before the start of the test, the field worker will demonstrate how to do the standing broad jump. Each child will then stand behind a straight line and jump as far forward as possible with both legs. He/she will have 2 tries (with a 30 sec rest in between) and the longest jump will be recorded (to the nearest 1 cm). The distance of the jump will be measured from the starting line to the heel of the most back foot.

(V) Mid body strength will be measured with the trunk extension test. The purpose of this test is to determine the range of motion when the back is negatively arched from the prone position. Flexibility and strength imbalances in the abdominal and lower back region are associated with back pain. This examination allows us to test the power capability of the trunk muscles. Gym mats and a measuring device, for instance a ruler, are required to administer this test. The study participant being tested lies on the mat in a prone and facedown position. Toes are pointed and hands are placed under the thighs. A coin or other marker is placed on the floor in line with the study participant's eyes. During the movement, the proband's focus should not move from the coin or marker. The study participant lifts the upper body off the floor, in a very slow and controlled manner, to a maximum height of 30 cm. The head should be maintained in a neutral and straight alignment with the spine. The position is held long enough to allow the tester to place the ruler on the floor in front of the person being tested and determine the distance from the floor to the proband's chin. The standardization allows two trials whereas the highest score is recorded. It should be noted that it is not advisable to encourage the study participant to hyperextension (30 cm and more) and not to motivate to do ballistic and bouncing movements.

(VI) Upper body strength will be determined with the grip strength test. The TKK® dynamometer will be used for this test. Before the start of the test, the hand span (distance from the tip of the thumb to the tip of the little finger) of the child's dominant hand will be measured (to the nearest 0.5 cm) and the grip span on the dynamometer adjusted accordingly [42, 43]. The field worker will also demonstrate how to grip the dynamometer to the child. The child should stand straight, yet relaxed and grip the dynamometer with the arm fully extended. During this time, no other parts of the body should touch the dynamometer. Each child will then have 2 tries

(with a 30 sec rest in between) to grip the dynamometer as hard as possible with their dominant hand and the maximum reading (measured to the nearest 0.5 kg) will be recorded.

(VII) Flexibility is an important component of health and a lack of flexibility could be of concern in obese children. We will rely on the sit-and-reach Test (SRT) for an indication of flexibility. This test measures flexibility of the hamstring muscles (back of the thigh) and, to a minor extent, the lower back muscles. The test protocol is as follows. The study participant is kindly asked to sit on the floor with direct contact to the wall and stretched legs. One should lean the back and head against the wall with the foot against the sit-and-reach box. Then, the hands should be placed over each other and stretched forward while the back should remain on the wall. The distance between the fingertips and the box is the starting point. With the hips bent as far as possible forward, the fingers should move as far as possible along the ruler to the front.

(VIII) For the measurement of coordination skills and speed strength of the leg muscles, we aim to complete the "jump sideways" test. The task for the participant is to jump laterally with both legs at the same time as many times as possible within 15 sec across a wooden bar. A field investigator will demonstrate the test in advance and five jumps can be practiced.

(IX) The physical activity questionnaire for children (PAQ-C) is a self-administered, 7-day recall instrument. It was developed to assess self-reported levels of general physical activity throughout the elementary school year for students, aged 8 to 14 years. The PAQ-C can be administered in a classroom setting and provides a summary physical activity score derived from nine items, each scored on a 5-point scale. Estimated completion time is 20 min per questionnaire.

Cognitive performance

(I) The AGTB 5-12 memory test is suitable for the measurement of the working memory of children aged 5 to 12 years. The AGTB 5-12 test will be recorded with 12 subtests, the three components of working memory: central executive, phonological and visual-spatial working memory. The AGTB 5-12 test is adaptive, that means that the difficulty level of the tasks will be adapted to both the actual and developmental age of the children and increased or decreased during the execution of the tasks depending on the performance. The instructions concerning all tasks are specified acoustically via loudspeaker and the test is performed on a computer. Direct evaluation is possible after performing the last task of the test, as the complete analysis for all performed subtests and the three working memory components can be seen at the end.

(II) The d2 test will be employed to measure cognitive performance. This test of attention is one of the most widely used measures of attention, particularly visual attention, in Europe and the USA [44]. The d2 paper-and-pencil version, which can be performed in a group setting, assesses several dimensions of cognitive performance: (i) total number of items processed (TN), a highly reliable measure of processing speed; (ii) percentage of errors (E%), measuring the qualitative aspects of performance; and (iii) the total number of items processed minus errors (TN-E), as an indication of the implications of the combined speed and accuracy scores for attentional and inhibitory control. Criterion, construct and predictive validity of the d2-test among children from the age of 9 years and older are well documented [45-47]. Moreover, the test offers an extensive list of norms, according to age, sex and education.

(III) The test of everyday attention for children (TEA-CH) is a test battery for measuring attention in children, in contrast to other tests, tasks have to be processed in parallel sometimes (e.g. a search task and the counting of acoustically given events). The test covers three areas of attention, namely selective attention, sustained attention and attentional control/shift.

(IV) The digit-span task (DST) is used to measure the working-memory's number storage capacity. Participants are presented with a series of digits (e.g., '8, 3, 4') and must immediately repeat them back. If they do this successfully, they are given a longer list (e.g., '9, 2, 4, 0'). The length of the longest list a student can remember is that person's digit span. While the study

participant is asked to enter the digits in the given order in the forward digit-span task, the participant needs to reverse the order of the numbers in the backward digit-span task.

(V) In cooperation with the schools, where every day good and reliable work is done, we want to record school test grades and discover reasons for school dropouts.

Questionnaire-based interviews for psychosocial health

To assess children's psychosocial health, the following paper-and-pencil questionnaires are applied:

(I) The KIDSCREEN-52 is used to assess children's physical and psychological well-being, moods and emotions, self-perception, autonomy, parent relation and home life, financial resources, peers and social support, school environment and bullying. The KIDSCREEN-52 has been proven to be a valid instrument to assess psychosocial health of children aged 8-18 years across various countries [48-50].

(II) The 13-item short version of the self-control scale (SCS) is used to assess individual differences in the capacity for self-control [51]. The human capacity of self-control has been described as one of the most powerful and beneficial adaptations of the human psyche [15, 51-54]. The exertion of self-control strengthens the relationship between the self and the environment, which is an important prerequisite for individuals' satisfaction with life, well-being and positive development [55, 56]. Evidence for the reliability and validity of the SCS has been demonstrated previously [51].

(III) The 9-item school-burnout inventory (SBI) [57] is applied to measure symptoms of school burnout. Salmela-Aro, Savolainen and Holopainen [58] showed that school burnout is able to predict subsequent depressive symptoms and that the influence of burnout on depressive symptoms is stronger than the impact of depressive symptoms on burnout. The SBI is a multifaceted instrument that includes three subscales (exhaustion at school [4 items], cynicism towards the meaning of school [3 items], and sense of inadequacy at school [2 items]). Answers are given on a 6-point Likert-scale ranging from 1 (completely disagree) to 6 (strongly agree). Evidence in support of the factorial and construct validity of the SBI has been provided previously [57-60].

6. Statistical analysis

6.1. Data collection and management

Types of data to be collected include:

(I) Quantitative data on anthropometric measurements, parasitological status, anaemia, hypertension and type 2 diabetes status and physical fitness, cognitive performance and psychosocial health levels;

(II) socio economic status and demographic data from each participant will be collected as potential confounders;

(III) outcome of the interventions (mid- and end-point) measured with the same parameters used at baseline of the intervention trial; and

(IV) qualitative data, based on the feasibility and acceptability of the interventions implemented.

The data will be double-entered, validated (EpiData) and merged into a single database (STATA). Statistical analysis will be performed using STATA and MAXQDA.

6.2. Methods for data analysis

Parasitological status will be assessed in terms of prevalence and infection intensity of individual parasitic species and the extent of multiparasitism. Clinical and anthropometric indicators and fitness and cognitive performance scores will be expressed as means, and questionnaire data pertaining to the psychosocial health will be expressed as percentages and treated as categorical data. All status and indicators will be compared between fit/non-infected and unfit/infected children and for the cluster randomized trial, the indicators will be compared between the intervention and control group. Changes in the parameters over time will also be examined.

To test the statistical significance of the effects of the different interventions on the parasitological status, clinical and anthropometric indicators, physical fitness, cognitive performance and psychosocial health levels:

(I) logistic regression with random effects will be used to compare binary data, such as parasitological status, clinical indicators and psychosocial health levels, between the intervention and control groups;
(II) linear regression with random effects will be used for numeric data, such as anthropometric measurements, physical fitness and cognitive performance scores, and haemoglobin concentration measurements; and
(III) multivariate analysis will also be done to account for confounders and interactions.

7. Ethical considerations

7.1. Ethical committee review

Ethical clearance for the study will be sought from the Ethics Committee Northwest and Central Switzerland (EKNZ) in Basel, Switzerland and from the following; ethics committees in South Africa:

(i) NMMU Health Sciences Faculty Research Committee;
(ii) NMMU Human Ethics Committee;
(iii) Eastern Cape Department of Education (for research done at schools); and
(iv) Eastern Cape Department of Health.

7.2. Duties of investigators

The proposed research will conform to international ethical and scientific standards as they are established in the Declaration of Helsinki, and promoted by the WHO and International Conference on Harmonization (ICH) Guidelines. The cluster randomized trial presented here will be registered in the current control trials register (http://www.controlled-trials.com/). Informed consent will be obtained from parents/guardians. The investigators will ensure the accuracy and completeness of the data reported in the study and will timely submit the required progress and final reports.

7.3. Confidentiality

Written informed consent will be obtained from the parents/guardians, whereas children will provide oral assent before the start of the study. Although the children included in the study will be encouraged to give their full participation, they can withdraw at any time without further obligation. The personal data of the children will be anonymized and all the data obtained will be used exclusively for scientific research. Records of the studies will remain confidential and to maintain confidentiality,

the PI will keep records in locked cupboards. After 5 years, these records will be destroyed. Data entered into computerized files will be accessible only by authorized investigators or medical personnel directly involved with the study.

7.4. Feed-back at the end of the study

At the end of the trial, the results will be communicated to the Department of Health in South Africa. All participating communities will be revisited at the end of the project and informed about the final study results. The PI, in collaboration with Swiss TPH, ISSW and NMMU will coordinate the international dissemination of the study results through presentations at national and international conferences and publications in the peer-reviewed literature (primarily open-access). We will also provide interventions for the control schools so that the whole community can benefit from this project.

7.5. Treatment

Appropriate treatment will be offered to all individuals of the included school classes participating in the study. Treatment will be administered by medical staff from the district hospital according to national treatment guidelines (e.g. 400 mg oral dose of albendazole for participants against soil-transmitted helminthiasis [61], and 40 mg/kg dose of praziquantel against schistosomiasis). Malaria will be treated with ACTs. Participants with persistent diarrhoea will receive oral rehydration solutions, while children with poor chronic conditions (e.g. type 2 diabetes and obesity), will be referred to a nearby health facility for treatment and care under an experienced medical personnel.

7.6. Risks and benefits of the study

There are no specific risks associated with this study. Submission of stool and urine samples by schoolchildren as study participants might be perceived as shameful. Fingerprick, in which a finger is pricked with a lancet to obtain a small quantity of capillary blood to test for anaemia (haemoglobin), diabetes and malaria through an RDT, is slightly an uncomfortable procedure, however produces no pain. Albendazole and praziquantel, widely used for preventive chemotherapy, might result in some adverse events, but these are usually mild and transient. The medical clinicians involved in this study will be prepared to treat the children in case of medical emergencies.

The proposed research will provide a comprehensive update on the status of communicable diseases and non-communicable chronic conditions in the selected communities nearby Port Elizabeth, South Africa. Since such data is currently not available in this area, there will be a need to generate more evidence. By linking them with the physical fitness, cognitive performance and psychosocial health of children, this wealth of information will help shed light on the true health consequences incurred by this potential dual burden of diseases and provide guidance for further health interventions to be implemented among school children in this area. Our intervention study will also highlight the applicability and scalability of the various health interventions in the study area. The results from this study will be summarized in manuscripts, which will be submitted for publication in peer-reviewed international literature, and whenever possible in open-access journals (e.g. BMC Public Health, Journal of Sports Sciences and the British Medical Journal). Results will also be presented at relevant national and international scientific meetings.

8. References

1. Murray, C.J.L., et al., *Disability-adjusted life years (DALYs) for 291 diseases and injuries in 21 regions, 1990-2010: a systematic analysis for the Global Burden of Disease Study 2010 (vol 380, pg 2197, 2012).* Lancet, 2013. **381**(9867): p. 628-628.
2. Lim, S.S., et al., *A comparative risk assessment of burden of disease and injury attributable to 67 risk factors and risk factor clusters in 21 regions, 1990-2010: a systematic analysis for the Global Burden of Disease Study 2010.* Lancet, 2012. **380**(9859): p. 2224-60.
3. Lozano, R., et al., *Global and regional mortality from 235 causes of death for 20 age groups in 1990 and 2010: a systematic analysis for the Global Burden of Disease Study 2010.* Lancet, 2012. **380**(9859): p. 2095-128.
4. Walter, C.M., *In-school physical activity patterns of primary school learners from disadvantaged schools in South Africa.* African Journal for Physical, Health Education, Recreation and Dance 17: 780-789., 2011.
5. Walter, C.M., Du Randt, R., *Socio-cultural barriers to physical activity among black isixhosa speaking professional women in the nelson mandela metropolitan municipality.* South African Journal for Research in Sport, Physical Education and Recreation 33: 143-155., 2011.
6. Steyn, K. and A. Damasceno, *Lifestyle and Related Risk Factors for Chronic Diseases*, in *Disease and Mortality in Sub-Saharan Africa*, D.T. Jamison, et al., Editors. 2006: Washington (DC).
7. Kimani-Murage, E.W., et al., *The prevalence of stunting, overweight and obesity, and metabolic disease risk in rural South African children.* BMC Public Health, 2010. **10**: p. 158.
8. Walter, C.M., Du Randt, R., Venter, D.J.L., *The physical activity and health status of two generations of Black South African professional women.* Health SA Gesondheid 16: 538., 2011.
9. Pillay, P., et al., *Real-Time Polymerase Chain Reaction for Detection of Schistosoma DNA in Small-Volume Urine Samples Reflects Focal Distribution of Urogenital Schistosomiasis in Primary School Girls in KwaZulu Natal, South Africa.* Am J Trop Med Hyg, 2014. **90**(3): p. 546-52.
10. Jardim-Botelho, A., et al., *Hookworm, Ascaris lumbricoides infection and polyparasitism associated with poor cognitive performance in Brazilian schoolchildren.* Trop Med Int Health, 2008. **13**(8): p. 994-1004.
11. Myer, L., R.I. Ehrlich, and E.S. Susser, *Social epidemiology in South Africa.* Epidemiol Rev, 2004. **26**: p. 112-23.
12. Draper, C.E., et al., *HealthKick: a nutrition and physical activity intervention for primary schools in low-income settings.* BMC Public Health, 2010. **10**: p. 398.
13. Kriemler, S., et al., *Effect of school based physical activity programme (KISS) on fitness and adiposity in primary schoolchildren: cluster randomised controlled trial.* BMJ, 2010. **340**: p. c785.
14. Bieri, F.A., et al., *A systematic review of preventive health educational videos targeting infectious diseases in schoolchildren.* Am J Trop Med Hyg, 2012. **87**(6): p. 972-8.

15. Baumeister, R.F., et al., *Self-regulation and personality: how interventions increase regulatory success, and how depletion moderates the effects of traits on behavior.* J Pers, 2006. **74**(6): p. 1773-801.
16. Muraven, M., R.F. Baumeister, and D.M. Tice, *Longitudinal improvement of self-regulation through practice: building self-control strength through repeated exercise.* J Soc Psychol, 1999. **139**(4): p. 446-57.
17. Oaten, M., Cheng, K., *Improvements in self-control from financial monitoring.* Journal of Economic Psychology, 2004. **28**: p. 487-501.
18. Gailliot, M.T., et al., *Increasing self-regulatory strength can reduce the depleting effect of suppressing stereotypes.* Pers Soc Psychol Bull, 2007. **33**(2): p. 281-94.
19. Oaten, M. and K. Cheng, *Longitudinal gains in self-regulation from regular physical exercise.* Br J Health Psychol, 2006. **11**(Pt 4): p. 717-33.
20. WHO, *Preventive chemotherapy in human helminthiasis: coordinated use of anthelmintic drugs in control interventions: a manual for health professionals and programme managers.* Geneva: World Health Organization., 2006.
21. Kish, L., *Sampling Organizations and Groups of Unequal Sizes.* Am Sociol Rev, 1965. **30**: p. 564-72.
22. Rowe, A.K., et al., *Design effects and intraclass correlation coefficients from a health facility cluster survey in Benin.* Int J Qual Health Care, 2002. **14**(6): p. 521-3.
23. Katz, N., A. Chaves, and J. Pellegrino, *A simple device for quantitative stool thick-smear technique in Schistosomiasis mansoni.* Rev Inst Med Trop Sao Paulo, 1972. **14**(6): p. 397-400.
24. Knopp, S., et al., *Diagnosis of soil-transmitted helminths in the era of preventive chemotherapy: effect of multiple stool sampling and use of different diagnostic techniques.* PLoS Negl Trop Dis, 2008. **2**(11): p. e331.
25. Utzinger, J., et al., *From innovation to application: social-ecological context, diagnostics, drugs and integrated control of schistosomiasis.* Acta Trop, 2011. **120 Suppl 1**: p. S121-37.
26. Sherkhonov, T., et al., *National intestinal helminth survey among schoolchildren in Tajikistan: prevalences, risk factors and perceptions.* Acta Trop, 2013. **126**(2): p. 93-8.
27. García, L.S., Bruckner, D.A., *Diagnostic Medical Parasitology.* Washington D.C.: ASM Press. 791 p., 2001.
28. Koga, K., et al., *A modified agar plate method for detection of Strongyloides stercoralis.* Am J Trop Med Hyg, 1991. **45**(4): p. 518-21.
29. Steinmann, P., et al., *Occurrence of Strongyloides stercoralis in Yunnan Province, China, and comparison of diagnostic methods.* PLoS Negl Trop Dis, 2007. **1**(1): p. e75.
30. Utzinger, J., et al., *Microscopic diagnosis of sodium acetate-acetic acid-formalin-fixed stool samples for helminths and intestinal protozoa: a comparison among European reference laboratories.* Clin Microbiol Infect, 2010. **16**(3): p. 267-73.
31. Coulibaly, J.T., et al., *Accuracy of urine circulating cathodic antigen (CCA) test for Schistosoma mansoni diagnosis in different settings of Cote d'Ivoire.* PLoS Negl Trop Dis, 2011. **5**(11): p. e1384.
32. Maltha, J., P. Gillet, and J. Jacobs, *Malaria rapid diagnostic tests in travel medicine.* Clin Microbiol Infect, 2013. **19**(5): p. 408-15.
33. WHO, *Growth reference 5-19 years.* Geneva: World Health Organization., 2007: p. pp.

34. Puder, J.J., et al., *Effect of multidimensional lifestyle intervention on fitness and adiposity in predominantly migrant preschool children (Ballabeina): cluster randomised controlled trial.* BMJ, 2011. **343**: p. d6195.
35. Niederer, I., et al., *Influence of a lifestyle intervention in preschool children on physiological and psychological parameters (Ballabeina): study design of a cluster randomized controlled trial.* BMC Public Health, 2009. **9**: p. 94.
36. Europe, C.o., *Testing physical fitness: Eurofit experimental battery.* Strasbourg: Council of Europe., 1983.
37. Meyer, U., et al., *Long-Term Effect of a School-Based Physical Activity Program (KISS) on Fitness and Adiposity in Children: A Cluster-Randomized Controlled Trial.* PLoS One, 2014. **9**(2): p. e87929.
38. Wanner, M., et al., *Effects of filter choice in GT3X accelerometer assessments of free-living activity.* Med Sci Sports Exerc, 2013. **45**(1): p. 170-7.
39. Gauderman, W.J., et al., *The effect of air pollution on lung development from 10 to 18 years of age.* N Engl J Med, 2004. **351**(11): p. 1057-67.
40. de Marco, R., et al., *Risk factors for chronic obstructive pulmonary disease in a European cohort of young adults.* Am J Respir Crit Care Med, 2011. **183**(7): p. 891-7.
41. Leger, L.A., et al., *The multistage 20 metre shuttle run test for aerobic fitness.* J Sports Sci, 1988. **6**(2): p. 93-101.
42. Espana-Romero, V., et al., *Hand span influences optimal grip span in boys and girls aged 6 to 12 years.* J Hand Surg Am, 2008. **33**(3): p. 378-84.
43. Ruiz, J.R., et al., *Hand span influences optimal grip span in male and female teenagers.* J Hand Surg Am, 2006. **31**(8): p. 1367-72.
44. Brickenkamp, R., Zillmer, E., *The d2 Test of Attention.* Seattle: Hogrefe & Huber Publisher., 1998.
45. Bates, M.E. and E.P. Lemay, Jr., *The d2 Test of attention: construct validity and extensions in scoring techniques.* J Int Neuropsychol Soc, 2004. **10**(3): p. 392-400.
46. Wassenberg, R., et al., *Development of inattention, impulsivity, and processing speed as measured by the d2 Test: results of a large cross-sectional study in children aged 7-13.* Child Neuropsychol, 2008. **14**(3): p. 195-210.
47. Gallotta, M.C., et al., *Effects of varying type of exertion on children's attention capacity.* Med Sci Sports Exerc, 2012. **44**(3): p. 550-5.
48. Ravens-Sieberer, U., et al., *KIDSCREEN-52 quality-of-life measure for children and adolescents.* Expert Rev Pharmacoecon Outcomes Res, 2005. **5**(3): p. 353-64.
49. Hong, S.D., et al., *The KIDSCREEN-52 quality of life measure for children and adolescents (KIDSCREEN-52-HRQOL): reliability and validity of the Korean version.* J Korean Med Sci, 2007. **22**(3): p. 446-52.
50. Ravens-Sieberer, U., et al., *The KIDSCREEN-52 quality of life measure for children and adolescents: psychometric results from a cross-cultural survey in 13 European countries.* Value Health, 2008. **11**(4): p. 645-58.
51. Tangney, J.P., R.F. Baumeister, and A.L. Boone, *High self-control predicts good adjustment, less pathology, better grades, and interpersonal success.* J Pers, 2006. **72**(2): p. 271-324.
52. Moffitt, T.E., et al., *A gradient of childhood self-control predicts health, wealth, and public safety.* Proc Natl Acad Sci U S A, 2011. **108**(7): p. 2693-8.
53. Duckworth, A.L., *The significance of self-control.* Proc Natl Acad Sci U S A, 2011. **108**(7): p. 2639-40.

54. Hagger, M.S., Wood, C., Stiff, C., Chatzisarantis, N.L.D. , *The strength model of self-regulation failure and health-related behavior.* Health Psychology Review, 2009. **3**: p. 208-238.
55. Duckworth, A.L., Grant, H., Loew, B., Oettingen, G., Gollwitzer, P.M., *Self-regulation strategies improve self-discipline in adolescents: Benefits of mental contrasting and implementation intentions.* Educational Psychology, 2010. **31**: p. 17-26.
56. Miller, H.V., J.C. Barnes, and K.M. Beaver, *Self-control and health outcomes in a nationally representative sample.* Am J Health Behav, 2011. **35**(1): p. 15-27.
57. Salmela-Aro, K., Kiuru, N., Leskinen, E., Nurmi, J.-E., *School-Burnout Inventory (SBI). Reliability and validity.* European Journal of Psychological Assessment, 2009. **25**: p. 48-57.
58. Salmela-Aro, K., H. Savolainen, and L. Holopainen, *Depressive symptoms and school burnout during adolescence: evidence from two cross-lagged longitudinal studies.* J Youth Adolesc, 2009. **38**(10): p. 1316-27.
59. Salmela-Aro, K., N. Kiuru, and J.E. Nurmi, *The role of educational track in adolescents' school burnout: a longitudinal study.* Br J Educ Psychol, 2008. **78**(Pt 4): p. 663-89.
60. Salmela-Aro, K. and L. Tynkkynen, *Gendered pathways in school burnout among adolescents.* J Adolesc, 2012. **35**(4): p. 929-39.
61. Keiser, J. and J. Utzinger, *Efficacy of current drugs against soil-transmitted helminth infections: systematic review and meta-analysis.* JAMA, 2008. **299**(16): p. 1937-48.
